# Breast carcinoma cells re-express E-cadherin during mesenchymal to epithelial reverting transition

**DOI:** 10.1186/1476-4598-9-179

**Published:** 2010-07-07

**Authors:** Yvonne L Chao, Christopher R Shepard, Alan Wells

**Affiliations:** 1 Department of Pathology, Pittsburgh VAMC and University of Pittsburgh, Pittsburgh, PA, 15213, USA

## Abstract

**Background:**

Epithelial to mesenchymal transition (EMT), implicated as a mechanism for tumor dissemination, is marked by loss of E-cadherin, disruption of cell adhesion, and induction of cell motility and invasion. In most intraductal breast carcinomas E-cadherin is regulated epigenetically via methylation of the promoter. E-cadherin expression is therefore dynamic and open to modulation by the microenvironment. In addition, it has been observed that metastatic foci commonly appear more differentiated than the primary tumor, suggesting that cancer cells may further undergo a mesenchymal to epithelial reverting transition (MErT) in the secondary organ environment following the EMT that allows for escape.

**Results:**

We first examined E-cadherin expression in primary breast tumors and their corresponding metastases to liver, lung and brain and discovered that 62% (10/16) of cases showed increased E-cadherin expression in the metastases compared to the primaries. These observations led to the question of whether the positive metastatic foci arose from expansion of E-cadherin-positive cells or from MErT of originally E-cadherin-negative disseminated cells. Thus, we aimed to determine whether it was possible for the mesenchymal-like MDA-MB-231 breast cancer cells to undergo an MErT through the re-expression of E-cadherin, either through exogenous introduction or induction by the microenvironment. Ectopic expression of full-length E-cadherin in MDA-MB-231 cells resulted in a morphological and functional reversion of the epithelial phenotype, with even just the cytosolic domain of E-cadherin yielding a partial phenotype. Introduction of MDA-MB-231 cells or primary explants into a secondary organ environment simulated by a hepatocyte coculture system induced E-cadherin re-expression through passive loss of methylation of the promoter. Furthermore, detection of E-cadherin-positive metastatic foci following the spontaneous metastasis of MDA-MB-231 cells injected into the mammary fat pad of mice suggests that this re-expression is functional.

**Conclusions:**

Our clinical observations and experimental data indicate that the secondary organ microenvironment can induce the re-expression of E-cadherin and consequently MErT. This phenotypic change is reflected in altered cell behavior and thus may be a critical step in cell survival at metastatic sites.

## Introduction

Breast cancer is the most frequently diagnosed cancer in women, and it is the second leading cause of cancer death in women of all ages [1]. Intraductal carcinoma, which originates from the epithelial cells lining the mammary ducts, is the most common type of breast cancer. Metastasis occurs via a series of sequential steps, during which the cells acquire an amoeboid-like phenotype, become motile, disseminate, and colonize distant sites of the body, which in breast cancer are most commonly liver, lung, bone, and brain. The stages of this transformation are similar to the stages of the developmental process known as epithelial to mesenchymal transition (EMT) [2]. Much of the current literature supports the idea that EMT is the key mechanism by which tumor cells gain invasive and metastatic ability, as EMT enables separation of individual cells from the primary tumor mass as well as promotes migration [3,4]. After undergoing EMT, thereby enabling access to hematogenous or lymphatic routes of dissemination, tumor cells can extravasate into secondary organs and establish micrometastases. We have hypothesized that EMT is reversible and that a reversion back towards the epithelial phenotype may occur at the secondary metastatic site (MErT). A similar reversion occurs in development when neural crest cells undergo a transient EMT followed by a permanent MET to generate tissues such as kidney epithelia [5]. A few studies have charted switches between EMT and MET phenotypes throughout malignant progression such as in colorectal cancer [6], bladder cancer [7], and ovarian cancer [8]. The phenotypic plasticity observed in these cases is unlikely to be generated by the acquisition of permanent genetic insults, suggesting that the microenvironment is capable of inducing epigenetic changes.

Numerous extracellular signals such as growth factors and stromal signals, and stressors such as hypoxia and ROS have been implicated in the induction of EMT [9]. However, at the core of the transition between an epithelial and a mesenchymal phenotype is the loss of E-cadherin expression. E-cadherin is a classical member of the cadherin family, whose extracellular domain facilitates homotypic intercellular adhesions while the cytosolic tail assembles catenins and other signaling and scaffolding molecules at the membrane to link to the actin cytoskeleton [10,11]. E-cadherin-mediated cell-cell adhesions limit cell motility and establish apical-basal polarity. The loss of E-cadherin expression and disassembly of E-cadherin adhesion plaques on the cell surface enables tumor cells to disengage from the primary mass and move to conduits of dissemination [12]. This duality of functionalities--intercellular cohesion and regulation of intracellular signaling cascades--suggests that E-cadherin may impact multiple aspects of epithelial homeostasis.

Thus, E-cadherin expression is intimately connected to a cell's degree of epitheliality - in both morphology and migratory and invasive abilities. In cancer pathogenesis, E-cadherin expression is dynamically regulated via epigenetic mechanisms, specifically methylation of the promoter, providing tumor cells the plasticity to switch between EMT and MErT depending on the microenvironment [13]. Interestingly, it has been observed that metastases often resemble the epithelial-like phenotype of the primary tumor rather than the mesenchymal phenotype observed at the invasive front. In addition, several pathological studies, including the one conducted herein, have observed increased E-cadherin expression in metastases compared to aberrant or loss of expression in the primary tumors, further challenging the notion that EMT is irreversible and suggesting that E-cadherin may be involved in MErT at the metastatic site [14,15]. However, one limitation of these pathological studies is that it is impossible to determine whether these E-cadherin-positive metastases result from the rare escape and expansion of epitheloid carcinoma cells, such as in the cell cooperativity model, or whether they arise from a mesenchymal-like cell that has undergone a phenotypic reversion back to a more differentiated phenotype, as we hypothesize [16,17].

Therefore, we aimed to experimentally determine whether it was possible for the mesenchymal-like MDA-MB-231 breast cancer cells to undergo an MErT through the re-expression of E-cadherin, either through exogenous introduction or through induction by the microenvironment. Ectopic expression of E-cadherin in MDA-MB-231 cells resulted in a reversion back to some degree of the epithelial phenotype, particularly with respect to morphology and functional suppression of migration and invasion. Furthermore, introduction of breast cancer cells and primary explants into a secondary organ environment led to the passive loss of methylation of the E-cadherin promoter and re-expression of this cell-cell adhesion molecule, demonstrating a mechanism for this reversion of EMT. *In vivo *experiments in mice revealed similar results in lung metastases, suggesting that re-expression of E-cadherin may be a critical step in metastatic colonization of not only the liver but lung as well.

## Results

### E-cadherin is expressed in distant metastases of E-cadherin-negative primary tumors

**Figure 1:**
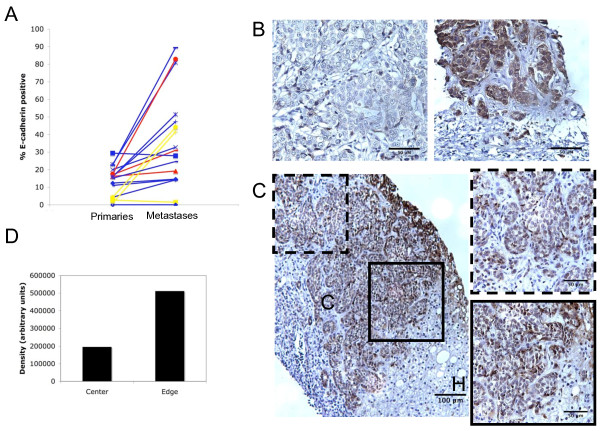
**E-cadherin expression is increased in metastases compared to primary tumors**. A) Percentage of E-cadherin-positve cells is increased in metastases compared to the primary tumors. Organ sites of metastases are organized by color: liver (red), lung (blue), and brain (yellow) B) Example of a case showing strong expression of E-cadherin in the metastasis (right) compared to negative expression in primary (left). C) Heterogeneous expression of E-cadherin in the center (dashed inset) versus edge (solid inset) of a liver metastasis. "C" denotes tumor and "H" denotes hepatocytes. D) Quantification of E-cadherin staining in the center and edge of the liver metastasis.

Loss of E-cadherin expression in the primary tumor is correlated with poor prognosis and survival [14,18]. A few studies have examined E-cadherin expression in the primary tumor and distant metastases, but the cases analyzed in these studies included metastases to lymph nodes or uncommon sites of breast cancer metastasis [15]. To conduct our own survey focusing on metastases to the most common sites, we obtained specimens of primary tumors and the corresponding metastases from 16 patients with infiltrating ductal carcinoma. Metastatic sites from which tissue was obtained included the lung (10 cases), liver (3), and brain (3). Both primary tumor and metastases were immunostained for E-cadherin. E-cadherin positive cells were counted based on high intensity membrane or cytoplasmic staining. Percentage of E-cadherin positivity was calculated as the number of E-cadherin-positive cells over the total number of cancer cells in each field (Additional file 1). Overall, 62% (10 of 16) cases showed increased E-cadherin expression in the metastases compared to the primary tumors (Figure 1a), with this being consistent across the various sites; 66% (2/3) of liver metastases, 66% (2/3) of brain metastases, and 60% (6/10) of lung metastases exhibited increased E-cadherin expression. There was no correlation between hormone receptor or Her2/neu status and E-cadherin expression. In some cases, closer examination of the specimens revealed striking differences of E-cadherin expression between the primary tumor and the metastasis, with the primary tumor wholly negative and the metastasis mostly positive for E-cadherin expression; one such liver metastasis is shown (Figure 1b). E-cadherin expression within both the primary tumors and the metastases was often heterogeneous, which was accounted for by quantifying areas of the tumor that best approximated the heterogeneity observed in the sample. However, even with this heterogeneity the levels of E-cadherin positivity were increased in the metastases (Additional file 1). In addition, the sizes of metastases ranged greatly, from micrometastases less than 1 mm to macrometastases greater than 2 cm in diameter. The trend appeared likely that heterogeneity of E-cadherin expression was positively correlated with tumor size; however, due to our small sample size we were unable to statistically assess such a correlation.

Of interest, E-cadherin expression in the metastases did not appear to be random. Shown is a liver metastasis demonstrating increased expression at the hepatocyte-cancer cell interface and decreased expression centrally, suggesting that E-cadherin is directly regulated by hepatocyte interactions (Figure 1c). Quantification of staining intensity confirmed an increase in E-cadherin expression in the area outlined by the solid inset compared to the area outlined by the dashed inset located further away from hepatocytes (Figure 1d). E-cadherin staining in the tissue samples is observed both at the membrane and in the cytoplasm, as autocrine EGFR signaling generally present in breast cancer drives E-cadherin internalization [19,20]. This overview of a small number of paired specimens provides insights into whether MErT is possible. If metastases are the result of expansion of a clonal population of cells originating from a primary tumor cell that has undergone EMT, then one would expect metastases to be E-cadherin-negative unless this phenotype is plastic. The finding of E-cadherin-positive metastases suggests that non-EMT cells can establish metastases or that MErT at the metastatic site can occur.

### Ectopic expression of E-cadherin partly reverts breast cancer cells towards an epithelial phenotype

**Figure 2:**
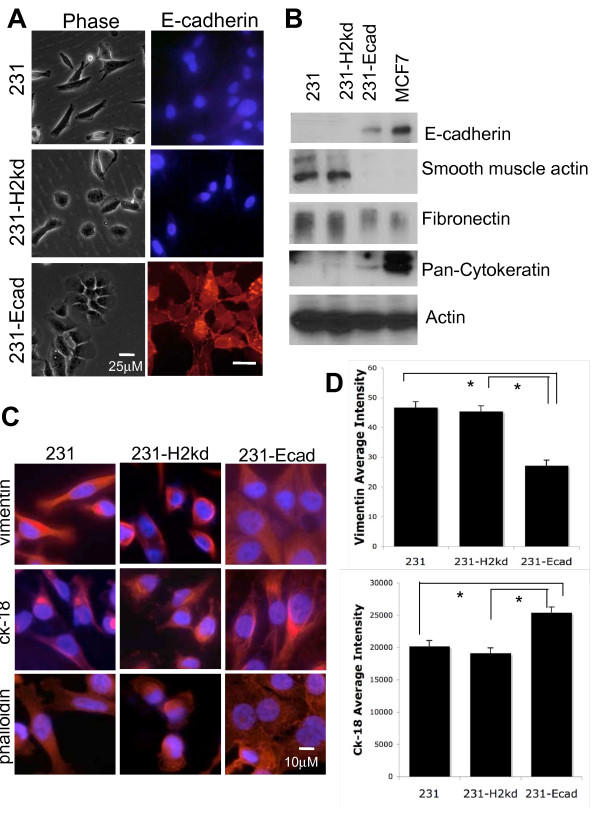
**E-cadherin expression alters cell morphology**. A) Cell morphology as examined by phase contrast microscopy (left column) and E-cadherin expression (red) as detected by immunofluorescence (right column) B) Immunblot analysis illustrates ectopic expression of E-cadherin in 231-Ecad cells as well as expression of various epithelial and mesenchymal markers in the E-cadherin mutants. C) Immunofluorescence of vimentin, cytokeratin-18 and actin cytoskeleton (rhodamine phalloidin). Shown are representative of at least three different assessments using one of two independent clones of each cell variant. D) Quantification of fluorescence using ImageJ, n = 20 cells, p < 0.05.

The finding of more prevalent E-cadherin expression in metastases compared to the paired primary tumors led to the question of whether the positive metastatic foci arose from expansion of E-cadherin-positive cells or from MErT of originally E-cadherin-negative cells. Thus, we aimed to determine whether it was possible for the mesenchymal-like MDA-MB-231 breast cancer cells to become more epithelioid following expression of E-cadherin. In MDA-MB-231 cells, E-cadherin expression is suppressed by methylation of the promoter. We stably transfected full-length E-cadherin driven by a CMV promoter and generated single cell clones (231-Ecad). In addition, because the possibility of intermediate EMT/MErT phenotypes has been proposed, we also stably transfected MDA-MB-231 cells with a construct composed of the intracellular and transmembrane domains of E-cadherin coupled to the class I major histocompatibility complex antigen (H-2kd) extracellular domain (231-H2kd). Such a construct was originally used to examine the contribution of internal E-cadherin signaling in the absence of E-cadherin-mediated intercellular interactions [21,22]. We postulated that expressing only the cytosolic tail of E-cadherin would allow for a partial MErT through the intracellular sequestration of adherens junction components and other effector proteins that is observed in epithelial cells but absent in mesenchymal cells. Immunoblot and immunofluorescence confirmed the exogenous expression of E-cadherin and E-cadherin-H2kd in MDA-MB-231 cells (Figure 2 and Additional file 2). 231-Ecad and 231-H2kd mutants display colocalization with the catenins at the membrane (Additional file 2b). E-cadherin expressing MCF7 breast cancer cells were used as a positive control. 231-Ecad cells exhibited cobblestone or cell-cell clustered morphology and formed cell contacts, which was not observed in control transfected MDA-MB-231 cells. 231-H2kd cells demonstrated a more flattened morphology that did not fully resemble either epithelial or mesenchymal phenotypes (Figure 2a). As expected, 231-H2kd cells did not form cell-cell contacts. It is important to note that this culture was performed at low cell density, so that cells were limited in establishing cell-cell connections. Thus, outside-in signaling mediated by E-cadherin was not necessary for the morphology change.

We next analyzed the expression of epithelial and mesenchymal markers in the various cell lines to monitor the penetrance of the epithelial/mesenchymal phenotypes. We evaluated the expression of a spectrum of cytokeratins including cytokeratin-18 (CK-18), the primary intermediate filament present in epithelial cells. Expression of vimentin, smooth muscle actin, and fibronectin were used as markers of the mesenchymal phenotype. Loss of cytokeratins and increased expression of vimentin, smooth muscle actin, or fibronectin have been shown to occur concurrently with EMT in adenocarcinomas [23]. The survey of these epithelial and mesenchymal markers revealed that 231-Ecad cells demonstrated decreased expression of smooth muscle actin, fibronectin, and vimentin and increased expression of cytokeratins (Figures 2b and 2c). Upregulation of N-cadherin has been observed in EMT, but because N-cadherin is not expressed in MDA-MB-231 cells this mesenchymal marker was not tested. 231-Ecad cells displayed increased cytokeratin-18 and decreased vimentin expression as assayed by immunofluorescence (Figure 2c). As epithelial and mesenchymal cells also differ in their cytosketelal architecture, phalloidin was used to visualize the actin cytoskeleton. Expression of the entire E-cadherin molecule (231-Ecad) provided a more epithelial-like reticular actin filament meshwork (Figure 2c). The persistence of mesenchymal markers and failure to fully express epithelial markers in 231-Ecad cells compared to the epithelial MCF7 cells suggests that MDA-MB-231 cells transfected with E-cadherin (either wild-type or cytosolic tail) still maintain some aspects of mesenchymal phenotype.

**Figure 3:**
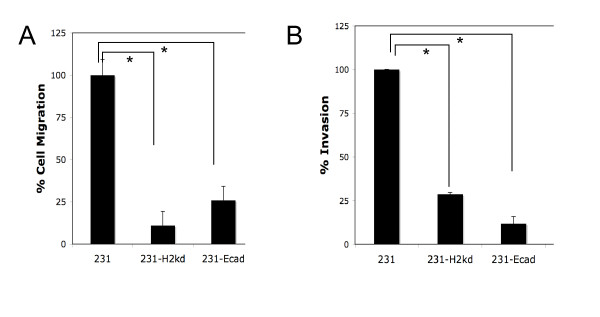
**E-cadherin expression suppresses migration (A) and invasion (B)**. Cell migration was analyzed using a scratch assay. Scratch closure was measured over a period of 24 hours and the fraction closure was quanitified by Metamorph software (n = 3). Invasion was measured in using a Matrigel invasion assay in which cells were allowed to migrate through a Matrigel-coated transwell insert for a period of 24 hours. N = 3 in triplicate; mean ± s.e.m. Results shown are representative of one of two independent clones of each mutant.

Mesenchymal and epithelial phenotypes also confer functional behaviors on tumor cells. As such we tested the two key properties related to tumor escape enabled by EMT: migration and invasion. After an *in vitro *scratch assay, which measures migration, we observed that expression of full-length or the cytosolic region of E-cadherin resulted in suppressed migration almost down to low levels noted for the epithelial MCF7 cancer line (Figure 3a). Similar trends were observed in the Matrigel invasion assay, which integrates motility with other properties such as matrix remodeling to better recreate the movement through bioactive matrices that defines tumor invasion. The invasive ability of both 231-Ecad and 231-H2kd cells was suppressed compared to MDA-MB-231 cells (Figure 3b). That suppression of migration and invasiveness were observed in 231-H2kd cells in the absence of changes in expression in the marker genes suggests that these functional behaviors may be independent of a mesenchymal to epithelial transition. While 231-H2kd cells may be similar to wildtype 231 in terms of mesenchymal and epithelial gene expression, β-catenin localization differed (Additional file 2); while 231 cells exhibit cytoplasmic distribution of β-catenin, 231-H2kd cells localize α-catenin, β-catenin, and p120 to the cell membrane as do the epithelial counterparts 231-Ecad and MCF7 cells. As reported by other groups, this alteration alone is sufficient to account for the invasion suppressor phenotype [24].

In summary, these results indicate that expression of exogenous E-cadherin (wild-type or cytosolic tail) in MDA-MB-231 cells results in a morphological shift toward the epithelial end of the spectrum. The expression of both epithelial and mesenchymal markers in 231-Ecad and 231-H2kd cells demonstrate that these cells may not have undergone a complete MErT, but the migration and invasion assay data suggest that expression of the full-length and cytosolic domains of E-cadherin are sufficient to induce a more epithelial-like phenotype in terms of cell motility and invasiveness. Furthermore, suppression of invasion and migration in 231-H2kd was comparable to the suppression in 231-Ecad cells, indicating that changes to the localization of key signaling proteins during the mesenchymal to epithelial transition can have profound effects in mitigating the mesenchymal nature of an invasive cell.

### E-cadherin expression is induced by a secondary organ microenvironment

**Figure 4:**
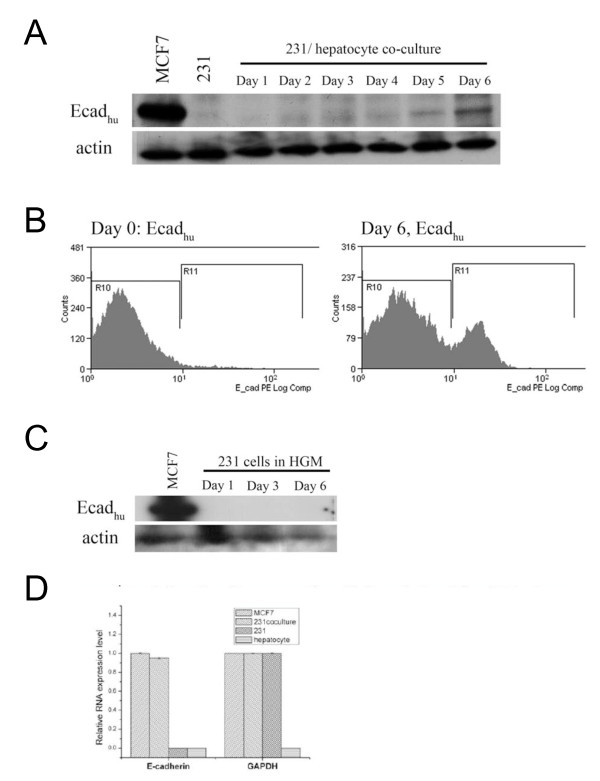
**Hepatocytes drive the re-expression of E-cadherin in MDA-MB-231 breast cancer cells**. A) Immunoblot of proteins lysates from MDA-MB-231/hepatocyte co-cultures using a human-specific antibody. B) Flow cytometry of the MDA-MB-231 population using a human-specific antibody shows a unimodal population on day 0 and a bimodal population on day 6. C) MDA-MB-231 cells do not express E-cadherin without hepatocytes. D) RT-PCR using human-specific primers of MDA-MB-231 cells after 6 days of co-culture with hepatocytes. Means (n = 4) ± s.d. Note that species-specific primers do not amplify E-cadherin or GAPDH from hepatocytes.

**Figure 5:**
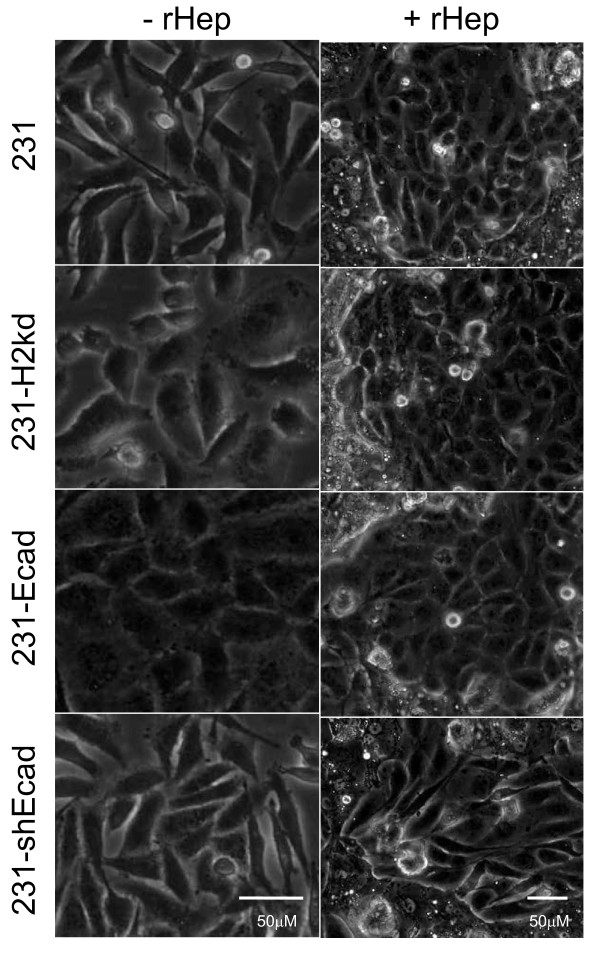
**Breast cancer cells cultured with hepatocytes revert to an epithelial morphology**. Phase contrast images of 231, 231-H2kd, 231-Ecad, and 231-shEcad breast cancer cells cultured with rat hepatocytes for 6 days.

Our previous results demonstrating E-cadherin expression in metastases suggested that a reversion to a more epithelial phenotype could occur at the metastatic site. We therefore hypothesized that a secondary organ microenvironment could induce re-expression of E-cadherin. To test this hypothesis, we cultured MDA-MB-231 cells with rat hepatocytes, as the liver is one of the main organs to which breast cancer cells metastasize. After 6 days of culture, expression of E-cadherin was detected using a human specific E-cadherin antibody (Figure 4a). Control experiments confirmed that the human-specific antibody did not cross-react with E-cadherin of rat origin, indicating that the E-cadherin was re-expressed by MDA-MB-231 cells (data not shown). Expression was also detected by flow cytometry (Figure 4b). Side and forward scatter as well as hepatocyte-specific autofluorescence gating were used to exclude the hepatocyte population. Flow cytometry analysis of MDA-MB-231 cells after 6 days of co-culture with hepatocytes formed a bimodal distribution, with 22.32% of cells forming a distinct population of E-cadherin positive cells. Culture of MDA-MB-231 cells in hepatocyte growth media alone did not result in re-expression, indicating that the re-expression is driven by hepatocytes (Figure 5c). Increased expression of E-cadherin mRNA was also detected by qRT-PCR (Figure 5d). After 6 days of culture with hepatocytes, MDA-MB-231 exhibited levels of E-cadherin transcript comparable to E-cadherin-positive MCF7 cells, while MDA-MB-231 cells cultured in the absence of hepatocytes presented undetectable mRNA levels. The fact that the E-cadherin mRNA level appears to be similar to that in MCF-7 cells despite lower protein levels is likely due to autocrine EGFR signaling driving E-cadherin internalization and degradation [15].

**Figure 6:**
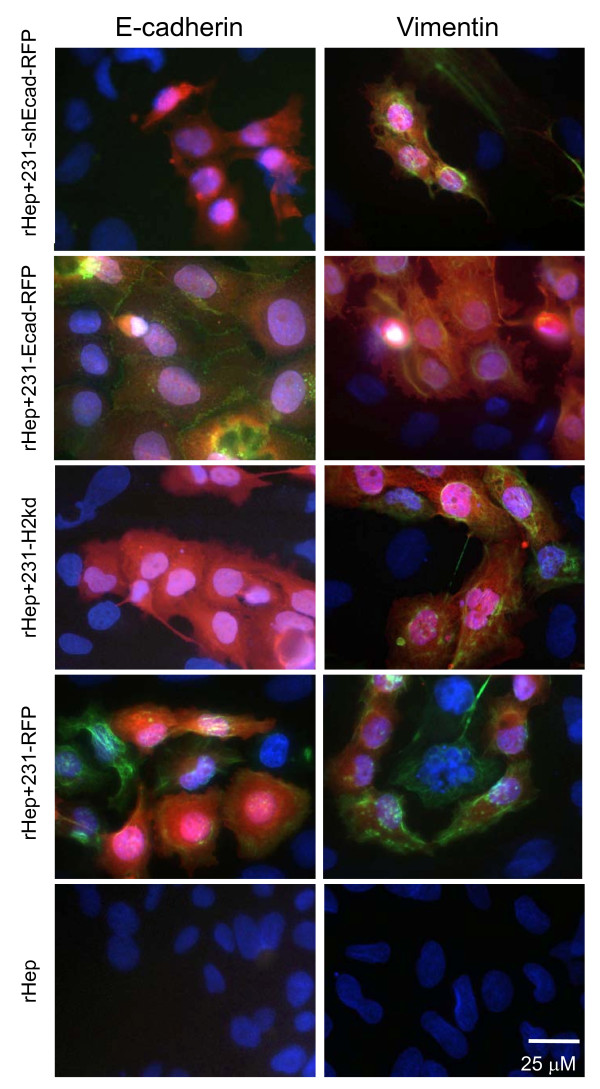
**Breast cancer cells culture with hepatocytes re-express E-cadherin but maintain vimentin A) Immunostaining of RFP-labeled breast cancer cells in hepatocyte coculture; E-cadherin (green), RFP (red), DAPI (blue) B) Immunostaining for vimentin (green), RFP (red), DAPI (blue)**. Shown are representative of at least three different assessments using at least two independent clones of each cell variant.

To prevent re-expression of E-cadherin in coculture and to validate that the changes noted were from E-cadherin and not another undefined co-expressed protein, we stably transfected MDA-MB-231 cells with an E-cadherin shRNA plasmid construct and generated single cell clones (231-shEcad). In addition, breast carcinoma cells were RFP-labeled to more easily discriminate cancer cells from hepatocytes in coculture. While MDA-MB-231, 231-H2kd, and 231-Ecad cells reverted to an epithelial clustered morphology following hepatocyte coculture, 231-shEcad cells remained fibroblastic (Figure 5). Immunofluorescence confirmed that the shRNA construct prevented re-expression of E-cadherin (Figure 6, left column). To evaluate whether MErT occurs following E-cadherin re-expression, cocultures were immunostained for the mesenchymal marker vimentin. Just as expression of mesenchymal markers persisted in 231-Ecad cells, E-cadherin re-expression in coculture did not completely suppress expression of vimentin (Figure 6, right column). However, vimentin expression appeared more heterogeneous, with some cells expressing more than others. It is important to note that compared to 231-Ecad cells where E-cadherin was exogenously expressed, there may be other unexplored molecular changes in MDA-MB-231 cells following hepatocyte coculture besides E-cadherin re-expression.

**Figure 7:**
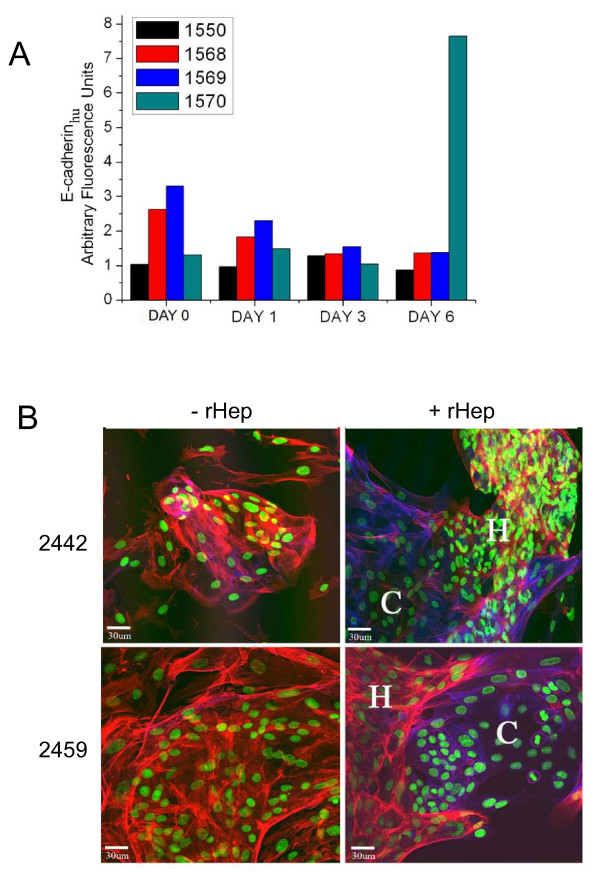
**A subset of primary breast carcinoma explants re-express E-cadherin when cocultured with primary hepatocytes**. A) Flow cytometry analysis of primary explants using a human-specific E-cadherin antibody. A fluorescence unit of 1 indicates that the fluorescence intensity was equal to the same gate performed without addition of antibody. B) Confocal microscopy of two positive explants. Explants (C), hepatocytes (H). Human-specific E-cadherin, blue; actin, red; nuclei, green.

As we demonstrated that it was possible for mesenchymally-transitioned carcinoma cells to revert to a more epithelioid phenotype, we next tested whether primary explants of human breast tumors could also re-express E-cadherin in hepatocyte coculture. Explants were obtained from breast tumors without current evidence of dissemination and cultured for at most 3 passages prior to experimentation. In total, four cocultured primary explants were assayed by flow cytometry and seven primary explants were analyzed by immunofluorescence following hepatocyte coculture. Analysis by flow cytometry indicated that although initially E-cadherin negative, one of the four explants tested expressed E-cadherin after coculture (Figure 7a). Similarly, tumor cells in two of seven explants that were originally E-cadherin negative, expressed robust and well-localized E-cadherin after 6 days of co-culture with the hepatocytes (Figure 7b). We were unable to ascertain the promoter methylation status in these cells due to the limited number and passage integrity of the primary cells; nonetheless, this line of evidence strongly suggests that primary human breast cancer cells may undergo similar molecular changes as MDA-MB-231 cells when cultured in a hepatic microenvironment.

### E-cadherin re-expression in the liver microenvironment is due to loss of promoter methylation

**Figure 8:**
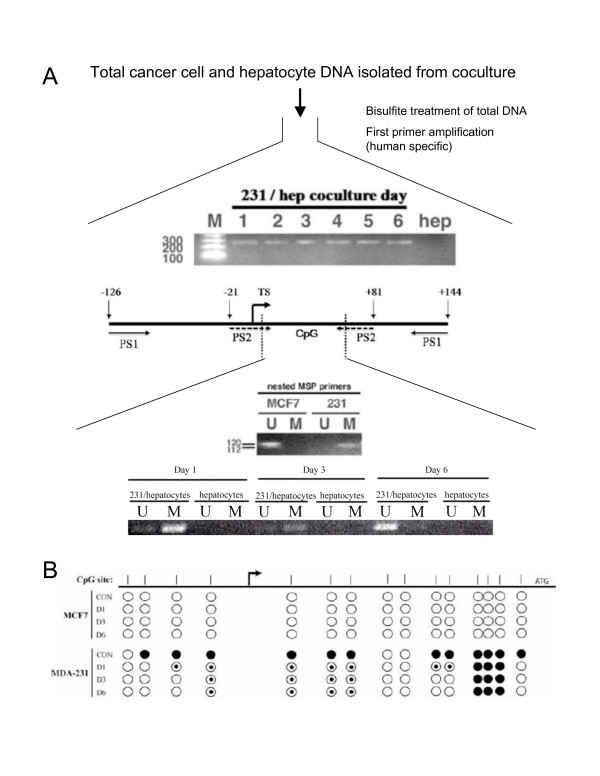
**Breast cancer cells lose methylation of E-cadherin promoter methylation following hepatocyte coculture**. A) Nested PCR method to detect methylation status of the E-cadherin promoter in a six day time course of hepatocye coculture. Above, bisulfite-treated DNA is amplified with primers that exclude CpG islands to amplify a 270 bp region independent of methylation status. Below, nested primers anneal to the 270 bp target to amplify a methylated (112 bp) or unmethylated (120 bp) fragment in the six day time course. MCF7 is used an unmethylated control. B) Bisulfite sequencing of CpG islands in the E-cadherin promoter. Figure adapted from Corn *et al *. CpG islands are indicated as vertical lines on map; each CpG island is represented a circle. MCF7, MDA-MB-231, and MDA-MB-435 were sequenced on days 1,3, and 5 coculture. Open circle, unmethylated CpG; closed circle, methylated CpG; filled circle, mixed quality values.

**Figure 9:**
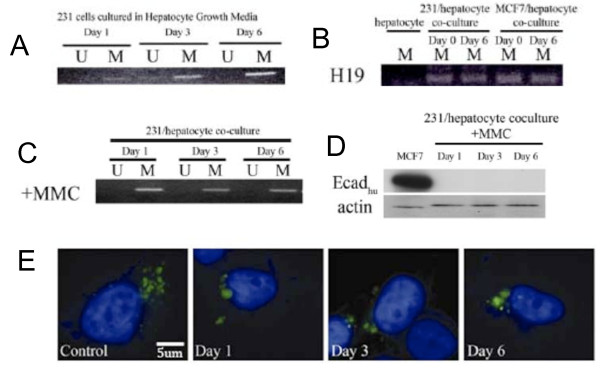
**Re-expression of E-cadherin follows a proliferation-dependent demethylation of the E-cadherin promoter**. A) MS-PCR of MDA-MB-231 cultured alone in hepatocyte growth media B) MS-PCR using human-specific primers that amplify the imprinted H19 gene. C) MS-PCR of E-cadherin promoter following addition of MMC D) Addition of MMC prevents E-cadherin re-expression at the protein level. E) The maintenance demethylase DNMT1 does not change in localization or intensity in MDA-MB-231 cancer cells when cocultured with hepatocytes. DNMT1, red; DAPI, blue.

In the absence of hepatocytes, E-cadherin expression in MDA-MB-231 cells is transcriptionally repressed by methylation of the E-cadherin promoter. Most intraductal breast carcinomas in which E-cadherin is downregulated also exhibit similar promoter hypermethylation [25]. Therefore loss of promoter methylation was examined as a possible mechanism for the re-expression of E-cadherin. We assayed a CpG island that was proximal to the E-cadherin transcription start site, whose methylation correlates inversely with E-cadherin expression [26]. Following coculture, total genomic DNA was isolated for methylation-specific PCR (MS-PCR) [27]. Species-specific primers were used to guarantee measurement of CpG methylation in only the human cancer cells and not rat hepatocytes. When human MDA-MB-231 cells were co-cultured with rat hepatocytes over a period of 6 days, the methylation status of the E-cadherin promoter region changed from a hypermethylated state to a hypomethylated state (Figure 8a). However, in the absence of hepatocytes, MDA-MB-231 cells remained hypermethylated (Figure 9a). To capture the dynamic loss of methylation of the CpG sites along the length of the E-cadherin promoter region, bisulfite sequencing was performed on MDA-MB-231 cells. MCF7 cells were used as an unmethylated control for E-cadherin promoter analysis. As expected, the promoter regions of the MDA-MB-231 cells were highly methylated before co-culture with hepatocytes, as denoted by the filled in circles of the control row. After coculture, much of the methylation was lost from these specific CpG islands (Figure 8b). Thus, the bisulfite sequencing validates our MS-PCR results and shows that E-cadherin promoter methylation decreases upon co-culture with hepatocytes, resulting in re-expression.

Because cancer cells are often globally hypomethylated, we evaluated whether the loss of methylation was specific to the E-cadherin promoter or the result of global hypomethylation. The *H19 *gene is a paternally imprinted gene whose methylation is modulated during gametogenesis and does not change after terminal differentiation of a cell line [28]. We performed bisulfite MS-PCR analysis on MDA-MB-231 cells before coculture and following 1,3, and 6 days of coculture with hepatocytes, examining a previously reported CpG site of *H19*. Evaluation of the data revealed that the average methylation of *H19 *remained unchanged at all time points indicating that global hypomethylation is not responsible for the changes observed at the E-cadherin promoter (Figure 9b).

Loss of promoter methylation can result from either a passive mechanism (lack of maintenance methylation subsequent to mitosis) or an active mechanism (enzyme-mediated excision), though there are currently no well-defined demethylases. The presence of intermediate stages of promoter methylation on day 3 and extended time period to unmethylated status (6 days) suggested a passive mechanism. To test whether the loss of methylation was dependent on proliferation of the cancer cells, we inhibited proliferation of the cancer cells with mitomycin-C. This treatment completely prevented loss of methylation of the promoter as demonstrated by MS-PCR (Figure 9c). Furthermore, addition of mitomycin-C also prevented re-expression of E-cadherin at the protein level (Figure 9d). Inhibition of DNA methyltransferases, which mediate CpG island methylation, could also account for loss of methylation. However, immunostaining for DNA methyltransferase DNMT1 showed neither decrease in expression nor change in nuclear localization (Figure 9e). Taken together, these data point to passive loss of methylation as the mechanism by which E-cadherin is re-expressed.

### E-cadherin re-expression occurs in vivo

**Figure 10:**
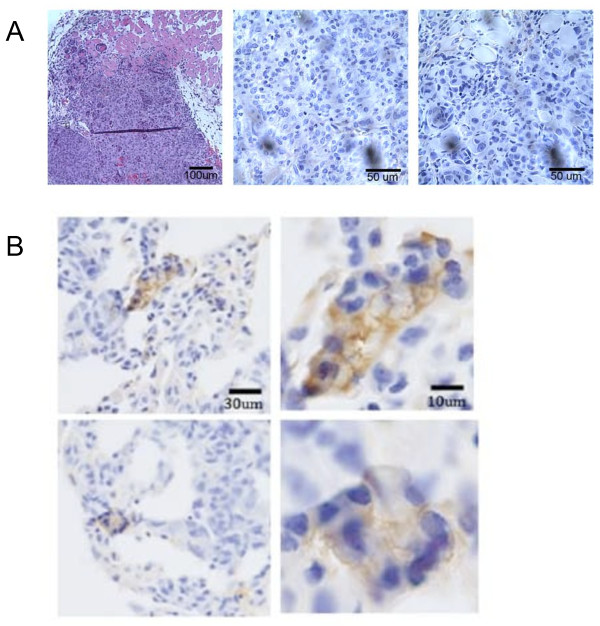
**E-cadherin positive metastatic foci originate from E-cadherin negative primary tumors**. A) Left, human MDA-MB-231 breast cancer cell xenograft in a mouse inguinal fat pad (H&E); middle, human-specific E-cadherin antibody indicates the absence of E-cadherin expression in the center of the primary tumor; right, absence of human-specific E-cadherin labeling at the periphery of the tumor. B) Micrometastases in the lung originating from the primary xenograft in *A*. Immunoperoxidase labeling of diseased portions of the mouse lung indicate the presence of human E-cadherin-positive MDA-MB-231 cancer cells; bottom adjacent.

To determine whether reversion of E-cadherin repression could be induced *in vivo*, we injected MDA-MB-231 cells into the mammary fat pads of mice. Mice were sacrificed after four weeks, to allow for dissemination from the primary tumor. Because MDA-MB-231 cells inoculated into the mouse mammary fat pad mainly metastasize to lung and not to liver when allowed to spontaneously metastasize, mice were examined for lung metastases by histopathological examination of the tissues. Our use of human breast cancer cells in a mouse host allowed for a human-specific E-cadherin antibody to discern the source of E-cadherin expression between the cancer cells and the epithelial mouse parenchyma. We first confirmed that the primary xenograft transplants in the inguinal mammary fat pads did not express E-cadherin (Figure 10a, left panel). There was no change in E-cadherin status of the invading cells in the primary xenograft, as we observed both the central and peripheral areas of the tumor to be devoid of E-cadherin as detected by immunoperoxidase staining (Figure 10a, middle and right panels). Two representative images of lung micrometastases less than 2 mm in diameter showed a markedly different pattern of E-cadherin expression. When immunoperoxidase labeling was performed on these sections, isolated islands expressing E-cadherin localized to the cell membrane were detected (Figure 10b). The human-specific antibody identified the disseminated MDA-MB-231 cells with robust E-cadherin expression, while not labeling the surrounding mouse lung tissue. Other fields of the same lung, unaffected and clear of metastatic lesions, did not display positive staining. Although we were unable to obtain metastases to the liver in the animal model, E-cadherin re-expression was observed in lung metastases in both the animal model and in clinical samples, suggesting that re-expression of E-cadherin may not be limited to the liver microenvironment.

## Discussion

Paget's seed and soil hypothesis has long postulated that cancer cells, or the "seeds", will only grow in a specific microenvironment, or "soil" [24,29-31]. Indeed, despite the fact that tumors are continually shedding cells, very few circulating tumor cells actually establish metastases, suggesting that post-extravasation survival is a crucial rate-limiting step [32]. The clinical observations that breast cancer displays a characteristic pattern of metastasis, specifically to the lung, liver, bone, and brain, indicate that these organs provide the most conducive microenvironment for metastatic growth. In addition, cancer cells themselves may exhibit an inherent gene signature predisposing them to homing to a particular organ site [14,18]. The precise environmental factors that enable the organotropism of metastases are yet to be fully discovered, but even less well known is why only a tiny fraction of circulating carcinoma cells form metastases.

Prior to extravasation, cancer cells must survive through invasion and emigration, anchorage-independent dissemination, and extravasation into the ectopic organ. These behaviors are thought to be conferred by molecular changes as a result of EMT. However, post-extravasation, cancer cells encounter a new set of challenges, notably integration within organ parenchyma and establishment of blood supply, which mesenchymal-like cells appear poorly equipped to handle. Despite the importance of EMT in promoting metastatic progression, there is mounting evidence that EMT is not an irreversible switch in cancer cell phenotype. Analysis of primary tumors and their corresponding metastases reveal that even though an EMT may have occurred to engender metastases, the phenotypes of the two can be strikingly similar. E-cadherin expression has been detected in lymph node and non-nodal metastases in carcinomas not limited to breast [33]. Re-expression of adhesion molecules could therefore be one way in which the secondary organ microenvironment promotes survival of metastatic cells as cadherin-cadherin engagement promotes activation of cell survival signaling pathways [19].

To ascertain whether these earlier reports of E-cadherin-expressing metastases held for intraductal breast carcinomas, we surveyed a small set of matched primary and metastatic tumors. Some 2/3 of metastases to the lung, liver, or brain expressed increased E-cadherin compared to the primary tumors, which largely exhibited aberrantly low to negative E-cadherin expression. Not all metastases exhibited high levels of E-cadherin expression, which is not surprising as metastases are known to evolve and give rise to further disseminations, suggesting that a second EMT may occur within more aggressive nodules.

Interestingly, E-cadherin expression even within metastases was heterogeneous, with increased E-cadherin expression seemingly correlated with proximity to normal parenchymal cells. This heterogeneity suggests that constant interaction with hepatocytes in liver may be necessary. Still, despite these observations, it was possible that these E-cadherin-positive tumor cells disseminated from the primary tumor as epithelioid cells and formed secondary metastatic lesions. Thus, we sought to provide proof-of-principle that cancer cells could be engineered to approach a mesenchymal-to-epithelial reverting transition by altering E-cadherin expression, either exogenously or via the microenvironment. We first hypothesized that we could engineer a MErT in MDA-MB-231 cells by expressing wild-type E-cadherin or by sequestering the E-cadherin-associated catenins with a non-binding E-cadherin construct. After transfecting the MDA-MB-231 cells with the cytosolic domain of E-cadherin linked to the MHC external domain, we saw that the dominant negative protein sequestered α-, β- and p120-catenins. The advantage of using this dominant negative is that the catenin signaling could be parsed from other activities of the extracellular domain of E-cadherin including cell adhesion through *trans*-ligation and EGFR *cis*-modulation [23,34,35]. While neither construct could completely revert MDA-MB-231 cells to an epithelial phenotype, expression of either construct resulted in morphological transformations and behavioral changes noted as suppression of migration and invasion. Our results also corroborate the findings of other studies focusing on the role of E-cadherin as a tumor or invasion suppressor [36-38].

When cultured in a hepatic microenvironment, MDA-MB-231 exhibited a similar reversion to an epithelial phenotype, both in morphology and E-cadherin re-expression. The nature of the signals that drive the reversion back to an epithelioid phenotype are not known and likely to be complex. Initial studies found that neither conditioned media nor hepatocyte-derived matrix could trigger E-cadherin re-expression in this breast carcinoma line, though the combination of the two was noted to lead to a weak re-expression of E-cadherin (data not shown). Re-expression secondary to loss of methylation of the E-cadherin promoter was also observed in the cell line MDA-MB-435 (Additional file 3), which is now considered to be a melanoma derivative, but is nonetheless useful as this neurectodermal lineage expresses E-cadherin as melanocytes but loses expression during melanoma progression [39]. Furthermore, this reversion is not likely unique to the liver microenvironement, based on the findings in human metastases and in our in vivo mouse model. Recently, we have found that lung parenchymal cells can drive E-cadherin expression in prostate tumor cells [40]. A recent study suggests that laminin-1 may be one component of the extracellular matrix that contributes to E-cadherin re-expression [15]. One key difference between our studies is the microenvironment used to induce E-cadherin re-expression in MDA-MB-231 cells. While Benton *et al *used a three-dimensional laminin-1 hydrogel, we chose to simulate a secondary organ microenvironment by culturing breast cancer cells with hepatocytes, thereby exposing them to hepatocyte-derived soluble factors and extracellular matrix. Their finding of DNMT1 downregulation as the mechanism for E-cadherin expression was not observed in our system (data not shown), suggesting that tissue architecture may induce MErT by alternative mechanisms. Thus, the search for this signaling 'cocktail' is likely to be complex and lies beyond the scope of the present communication.

That E-cadherin re-expression is caused by loss of methylation suggests a functional mechanism by which the microenvironment modulates the mesenchymal to epithelial phenotypic switch. E-cadherin is predominantly downregulated in carcinomas at the post-translational and/or transcriptional levels. Regulation of E-cadherin is therefore unique among tumor suppressors in which loss or mutation appears to be the rule, but this epigenetic regulation of E-cadherin allows for increased phenotypic plasticity. We have previously reported that prostate cancer cells cultured with hepatocytes also re-express E-cadherin, but as a result of inhibition of the EGF receptor signaling [24,35,41,42]. However, in breast cancers E-cadherin is silenced directly at the transcriptional level by promoter hypermethylation or indirectly through its transcriptional suppressors Snail, Slug, and Twist [43]. No differences in expression of these transcriptional suppressors were observed following hepatocyte coculture (data not shown). In MDA-MB-231 cells, representative of the basal subtype of infiltrating ductal carcinomas, the CpG islands in the promoter region most proximal to the E-cadherin initiation site are fully methylated, which exerts a profound effect on mesenchymal nature. Demethylation of these islands by the chemical agent 5-aza-deoxycytidine causes re-expression of E-cadherin and loss of invasive ability [44-47]. Coculturing of MDA-MB-231 cells with primary hepatocytes resulted in loss of methylation of the E-cadherin promoter and expression of E-cadherin mRNA and protein. We observed that the loss of methylation was dependent on the proliferation of the cancer cells. This finding was not unique to the breast carcinoma cells, as the MDA-MB-435 line also demonstrated loss of promoter hypermethylation upon coculturing with hepatocytes. Importantly, this loss of methylation was at least semi-specific and not global as the imprinted H19 gene remained methylated. The ubiquitous transcription factor Sp1 has been implicated in the regulation of methylation status by binding loci of hemimethylated DNA, protecting sequences from *de novo *methylation, preferential demethylation, or passive demethylation mechanisms [48]. Sp1 was necessary for loss of methylation in coculture (data not shown), strongly suggesting active signaling from the microenvironment.

The foundation of our findings rest on the epigenetic reversion observed when breast cancer cells are cocultured with primary hepatocytes. The epigenetic status of the primary tumor and disseminated metastases is most likely important, since primary tumors that have high E-cadherin levels have very little systemic disease [31,49], suggesting that the epigenetic reversion at distant secondary sites is also relevant. The xenograft model in which E-cadherin negative MDA-MB-231 cells formed E-cadherin-negative primary tumors in the mammary fat pads but E-cadherin-positive micrometastases and the finding that at least some E-cadherin-negative primary breast carcinoma cells can re-express this molecule support the idea that this reversion is possible. Furthermore, the xenograft experiment demonstrates that the molecular changes can occur in the secondary site. However, these experiments do not mean that all E-cadherin-positive metastases necessarily arise from the reversion of E-cadherin-negative cancer cells. Further molecular dissections and a much larger breast tumor survey, challenging due to the paucity of matched primary and non-nodal metastases, are needed to determine the extent of this MErT in early metastatic seeding.

The potential implications of E-cadherin re-expression and MErT are many. There are several possible outcomes or combinations of outcomes after a cell extravasates into a metastatic target tissue: apoptosis, dormancy, or sustained proliferation, with the latter appearing the rarest [50]. While E-cadherin typically mediates homotypic cell-cell adhesions, heterophilic ligation between different cell types has been documented [51-53]. Cancer cell adhesion has been shown to facilitate extravasation and colonization of distant organs [54,55]. Phenotypic reversion to epitheliality *in vivo *may therefore enhance the integration and survival of cancer cells at the metastatic site by cloaking the cancer cell with epithelioid-like characteristics, or may act to transmit mitogenic signals. E-cadherin expression has also been shown to suppress cell growth, which may account for the dormancy period between clinical presentation of metastases [56]. However, preliminary results in a parallel study reveal that one important survival advantage conferred by E-cadherin expression is increased resistance to cell death induced by chemotherapeutic agents such as camptothecin, doxorubicin, and taxol (data not shown). Cellular adhesion has long been implicated in intrinsic or acquired resistance of solid tumors to multiple anticancer therapeutics not restricted to chemotherapy [57,58]. The addition of E-cadherin function blocking antibodies sensitizes multicellular spheroids to treatment with various chemotherapeutic agents and E-cadherin-positive cells are more resistant to staurosporine-induced cell death than E-cadherin-negative breast cancer cells [20]. A similar survival advantage may be conferred when disseminated cells face apoptotic cytokines, thus providing a selective pressure that then confounds adjuvant therapies. The finding that E-cadherin re-expression and catenin sequestration can contribute to a MErT suggests that they may be appropriate therapeutic targets for preventing the establishment of metastases in breast cancer.

## Materials and methods

### Generation of cell lines

231-H2kd cells were generated using the *Myc*/His encoding H-2kd-E-cad dominant negative E-cadherin construct, a kind gift from Vizirianakis *et al *[15]. 231-H2kd cells were selected by FACS using the H-2kd (SF1-1.1) antibody (BD Pharmingen; San Jose, CA) and were maintained in 900 μg/ml G418 until used for experimentation. 231-Ecad cells were made by co-transfecting a plasmid encoding the E-cadherin full-length cDNA sequence (Open Biosystems) with the pcDNA 3.1 plasmid (Invitrogen) and cultured in 900 μg/ml G418 to select for stable transfectants. 231-shEcad cells were generated using an E-cadherin shRNA plasmid (Santa Cruz Biotechnology) and stable transfectants were selected using 5 ug/ml of puromycin and confirmed by RT-PCR. At least two single cell clones of each mutant were generated by selecting for resistance to G418 (231-H2kd and 231-Ecad) or puromycin (231-shEcad). Control clones transfected with pcDNA 3.1, DsRed2, and control shRNA were also generated and tested. Single cell clones of each mutant line were subsequently transfected with the DsRed2 plasmid vector and FACS sorted for RFP fluorescence for use in hepatocyte cocultures. In all cases the experiments were performed at least once with the different clones, rendering similar results.

### Cell culture and co-culture

MCF7, MDA-MB-231, and MDA-MB-435 cells were cultured in RPMI-1640 with 10% FBS as previously described [15]. Primary rat hepatocytes were isolated by collagenase perfusion and cultured as described previously [59] and plated onto collagen-coated 6-well plates at 60,000 cells/cm^2^. The following day, cancer cells were seeded onto the hepatocyte monolayer at 3,000 cells/cm^2 ^and cocultured for 6 days.

### Immunohistochemistry

Paraffin-embedded patient samples were obtained from Magee Womens Hospital. Sections underwent antigen retrieval in citrate solution and were incubated with E-cadherin primary antibody (Cell Signaling). Antigen staining was performed using DAB (Vector Laboratories) then counterstained with Mayer's hematoxylin. Secondary antibody alone served as a negative control and adjacent normal tissue served as an internal positive control. Images of three randomly-selected microscope fields of each sample were taken and the percentage of E-cadherin positive cancer cells was quantified as the number of E-cadherin positive cells over the total number of cancer cells in that image. Microscope fields shown were selected to account for the heterogeneity of each sample. Relative staining intensity of the liver metastasis was quantified using ImageJ software.

### Invasion assay

Invasive potential was determined *in vitro *by migration through an artificial ECM [26]. 2.5 × 10^4 ^cells were challenged in growth-factor reduced matrigel invasion chambers (BD Biosciences). Cells were seeded into the top chamber with serum-free media and media containing 10% serum was added to the lower chamber for the remainder of the assay. After 24 hours, the remaining cells and ECM in the top chamber were removed by cotton swab. Cells that invaded through the matrix to the bottom of the filter were then fixed and stained with DAPI and counted. Individual experiments were performed in triplicate.

### Scratch Assay

A monolayer of cells was grown to confluence in a 6-well plate and at experimental time zero a scratch was made in each well using a pipette tip. The well was imaged at time zero and again 24 hours later. Using Metamorph, a measurement was taken for how much the denuded area had filled in the 24-hour period.

### Xenografts

The Institutional Animal Care and Use Committee at the Veterans Affairs Hospital in Pittsburgh approved all animal procedures. Experiments were performed in 8 week old female athymic nude mice. One million MDA-MB-231 cells were injected into the right mammary fat pad; injection vehicle was the culture medium (0.2 mL/site). Mice were sacrificed 4-5 weeks after tumor cell implantation and the primary xenograft and lungs removed.

Xenograft and other harvested tissues were fixed in 4% buffered formalin and 4 μm thick paraffin sections underwent antigen retrieval for 5 min in 95°C 10 mM citrate solution in preparation for H&E and immunochemistry. With the use of the Mouse on Mouse Kit (Vector Labs, Berlingame, CA), positive labeling was confirmed by comparing serial sections incubated with the primary human-specific E-cadherin antibody (67A4 1:100; Santa Cruz Biotechnology, Santa Cruz, CA) or the biotinylated secondary antibody alone. Labeling was visualized with the Vectastain Elite kit (Vector Labs).

### Methylation Specific PCR and bisulfite sequencing

DNA was isolated from co-culture using the DNeasy Blood and Tissue Kit (Qiagen, Velencia, CA). 2000 ng of isolated DNA was subjected to bisulfite treatment using the EZ DNA Methylation Gold Kit (Zymo, San Diego, CA) per the manufacturer's specifications. MSP was performed in the way of Corn et al [60] or using the CpG WIZ E-cadherin Amplification Kit per the manufacturer's instructions (Millipore, Temecula, CA). Briefly, in the method of Corn, a nested PCR method was used, in which the first primer set generated a 270 bp fragment that was subsequently sequenced. The second round of PCR used either nested primers that were specific to either the unmethylated or methylated allele, which amplified the first CpG island after the transcription start site. The product size of the methylated reaction was 112 bp and 120 bp for the unmethylated.

MSP of H19 after bisulfite conversion was performed using the following primers: F 5'-TTA TAA AAT CGA AAA TTA CGC GCG A-3' R 5'-TTT TAG ATG ATT TTT GTG AAT TTT-3'. Cycling conditions were 95°C for 15 min, 35 cycles of 94°C for 1 min, 55°C for 1 min, and 72°C for 1 min with a final extension of 5 min at 72°C. All reactions were performed using Platinum Taq SuperMix (Invitrogen).

### Real-time quantitative PCR

RNA was isolated from hepatocyte-cancer cell co-cultures with the PureYield RNA Midiprep System (Promega, Madison, WI). cDNA was obtained with High Capacity cDNA RT Kit (Applied BioSystems, Foster City, CA). The human-specific TaqMan Gene Expression Assay Hs00170423_A1 CDHI probe was obtained from Applied Biosystems (Foster City, CA). Amplification and analysis in quadruplicate was run in an Applied Biosystems 7500 Real-Time PCR System. Relative values were normalized by using GAPDH levels as a reference using TaqMan Pre-Developed Human GAPDH Assay Reagent by Applied Biosystems.

### Immunoblotting, Immunofluorescence, and Flow Cytometry

Cell lysate proteins were resolved on 7.5% SDS-PAGE and and transferred to PVDF membranes. After blocking, membranes were incubated with primary antibodies against E-cdherin (Santa Cruz), pan cytokeratin (abcam), smooth muscle actin (Cal Biochem), fibronectin (Rockland Inc), GAPDH (Sigma) and actin (Sigma), followed by incubation with peroxidase-conjugated secondary antibodies and chemiluminescence detection.

For flow cytometry, co-cultures were non-ezymatically dissociated from the culture plates and vortexed into a single-cell suspension. The cells were fixed in 2% Paraformaldehyde for 30 minutes, permeabilized with 1% Triton for 3 minutes, and incubated with a PE-conjugated E-cadherin antibody (67A4) for 30 minutes. The mixed hepatocyte-cancer cell suspension was gated as to exclude hepatocytes using the appropriate SSC/FSC parameters. Data were collected on at least 10^6 ^cells in the appropriate SSC/FSC region.

Immunofluorescence was performed by overnight primary antibody incubation with E-cadherin (Santa Cruz), DNMT1 (Santa Cruz), DsRed (Santa Cruz), Alexa 488-phalloidin (Molecular Probes), cytokeratin-18 (abcam) or vimentin (abcam) followed by incubation with the appropriate fluorophore-labeled secondary antibody. Visualization was performed on an Olympus Fluoview 1000 confocal microscope (Olympus, Center Valley, PA).

### Primary explants

Polyclonal primary human tumor explants were obtained and cultured as previously reported [26]. Immunofluorescence labeling was performed as above.

### Statistical Analysis

All quantitative data are presented as mean ± sd obtained from independent experiments. p-value significance was determined using a two-tailed unpaired Student t-test, and set at 0.05 as a minimum. All images were representative of at least three independent observations.

## Competing interests

The authors declare that they have no competing interests.

## Authors' contributions

YC and CS performed experiments, analyzed data, and drafted the manuscript. AW participated in the design of the study, interpretation of data, and edited the manuscript. All authors read and approved the final manuscript.

## Supplementary Material

Additional file 1 **Tables of quantification of E-cadherin staining in primary and metastatic tumors of breast cancer patients**. Metastases are color-coded to mirror Figure 1A. Three microscope fields of each specimen were selected and quantified except when limited by the size of the sample. Click here for file

Additional file 2 **β- and p120-catenin are sequestered by the Ecad/H2kd fragment**. A) β- or p120-catenin, left panel, green; H2kd, middle panel, red; merge, right panel, yellow. In the merged images, the catenins colocalize with the H2kd molecules. B) β-catenin staining of 231, 231-Ecad and MCF7 cells. β-catenin is localized at the membrane in 231-Ecad and MCF7 cells but in the cytoplasm in 231 cells. C) Transfected MDA-231 cells express the H2kd fragment. When 231-H2kd whole cell lysates are probed with an H2kd antibody and immunoprecipitated, both beta- and p120 catenins coimmunoprecipitate as determined by western blot. Click here for file

Additional file 3 **MDA-MB-435 cells re-express E-cadherin**. A) Immunoblot of MDA-MB-435 cells cultured with hepatocytes for 6 days and probed with an E-cadherin antibody. B) Methylation-specific PCR of MDA-MB-435/hepatocyte samples reveals loss of methylation of the E-cadherin promoter. Click here for file
