# Supplementary material for: Breast carcinoma cells re-express E-cadherin during mesenchymal to epithelial reverting transition
Source: Mol Cancer. 2010 Jul 7;9:179. doi: 10.1186/1476-4598-9-179 (PMC2907333; doi:10.1186/1476-4598-9-179)
Supplement: Additional file 1 — Tables of quantification of E-cadherin staining in primary and metastatic tumors of breast cancer patients. Metastases are color-coded to mirror Figure 1A. Three microscope fields of each specimen were selected and quantified except when limited by the size of the sample. [file 1476-4598-9-179-S1.PDF]

	Primary Breast					Lung Metastasis				
**Case**	**Pos**	**Neg**	**Total**	**% Pos**	**AVG % Pos**	**Pos**	**Neg**	**Total**	**% Pos**	**AVG % Pos**
**1**	21	228	249	8.4	12.3	32	169	201	15.9	14.6
	36	136	172	20.9		33	158	191	17.3	
	20	245	265	7.5		24	203	227	10.6	
**8**	10	128	138	7.2	11.0	24	143	167	14.4	14.3
	4	106	110	3.6		23	159	182	12.6	
	23	81	104	22.1		39	206	245	15.9	
**9**	10	236	246	4.1	4.3	46	129	175	26.3	14.2
	8	252	260	3.1		14	155	169	8.3	
	14	225	239	5.9		17	193	210	8.1	
**13**	60	148	208	28.8	20.0	127	205	332	38.3	32.7
	47	124	171	27.5		75	247	322	23.3	
	8	210	218	3.7		120	208	328	36.6	
**14**	27	146	173	15.6	23.0	24	4	28	85.7	81.0
	42	111	153	27.5		20	5	25	80.0	
	38	108	146	26.0		17	5	22	77.3	
**16**	34	92	126	27.0	16.1	32	62	94	34.0	47.3
	10	137	147	6.8		46	30	76	60.5	
	31	181	212	14.6						
**18**	56	89	145	38.6	22.6	50	4	54	92.6	89.4
	17	109	126	13.5		93	30	123	75.6	
	20	108	128	15.6		33	0	33	100.0	
**20**	18	101	119	15.1	29.4	29	43	72	40.3	27.9
	45	65	110	40.9		12	70	82	14.6	
	25	53	78	32.1		25	62	87	28.7	
**23**	37	215	252	14.7	15.9	37	132	169	21.9	51.4
	18	262	280	6.4		102	92	194	52.6	
	86	239	325	26.5		106	27	133	79.7	
**26**	18	116	134	13.4	15.1	39	83	122	32.0	24.6
	27	122	149	18.1		38	102	140	27.1	
	17	108	125	13.6		17	99	116	14.7	

	Primary Breast					Liver Metastasis				
**Case**	**Pos**	**Neg**	**Total**	**% Pos**	**AVG % Pos**	**Pos**	**Neg**	**Total**	**% Pos**	**AVG % Pos**
**3**	3	195	198	1.5	16.2	48	229	277	17.3	19.2
	41	92	133	30.8		58	260	318	18.2	
						55	194	249	22.1	
**15**	23	124	147	15.6	17.5	94	20	114	82.5	82.9
	32	126	158	20.3		161	48	209	77.0	
	27	136	163	16.6		92	11	103	89.3	
**17**	91	86	177	51.4	17.1	65	144	209	31.1	
	0	100	100	0.0						
	0	100	100	0.0						

	Primary Breast					Brain Metastasis				
**Case**	**Pos**	**Neg**	**Total**	**% Pos**	**AVG % Pos**	**Pos**	**Neg**	**Total**	**% Pos**	**AVG % Pos**
**11**	6	222	228	2.6	2.6	0	100	100	0.0	1.4
	7	128	135	5.2		6	140	146	4.1	
	0	128	128	0.0		0	100	100	0.0	
**24**	0	100	100	0.0	4.2	38	24	62	61.3	44.2
	17	117	134	12.7		17	32	49	34.7	
	0	100	100	0.0		23	40	63	36.5	
**25**	4	149	153	2.6	0.9	48	97	145	33.1	41.6
	0	100	100	0.0		26	34	60	43.3	
	0	100	100	0.0		54	58	112	48.2	
