# Supplementary figures and images for: Breast carcinoma cells re-express E-cadherin during mesenchymal to epithelial reverting transition
Source: Mol Cancer. 2010 Jul 7;9:179. doi: 10.1186/1476-4598-9-179 (PMC2907333; doi:10.1186/1476-4598-9-179)

**A**

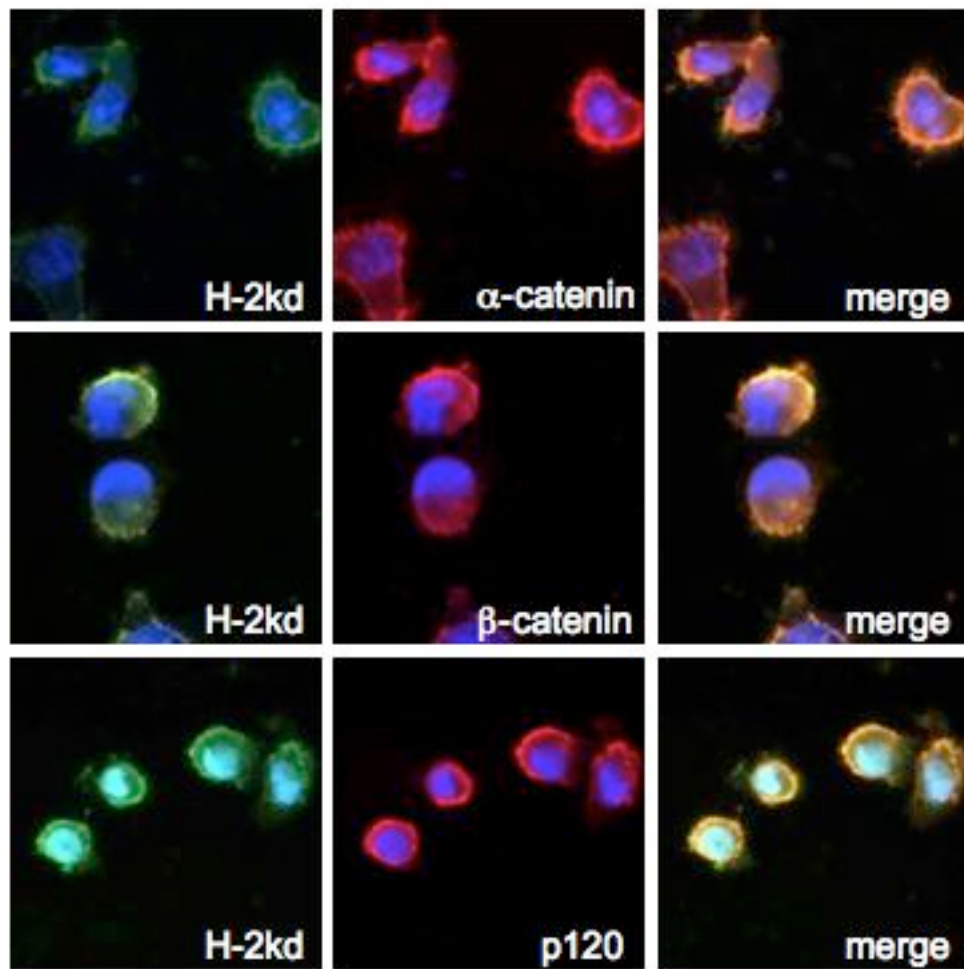

**B**

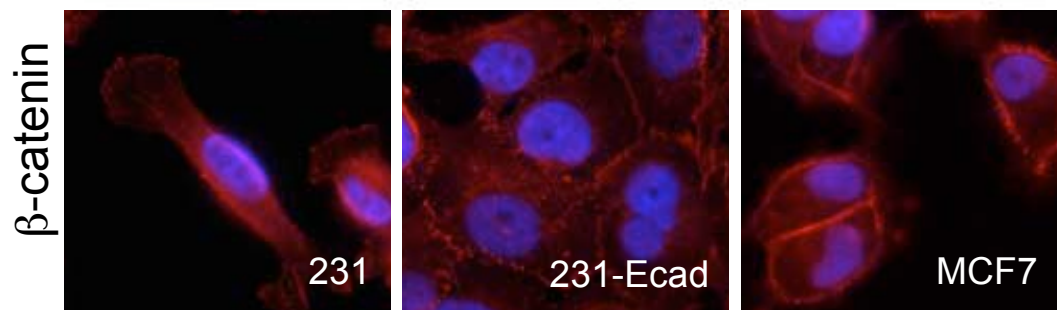

**C**

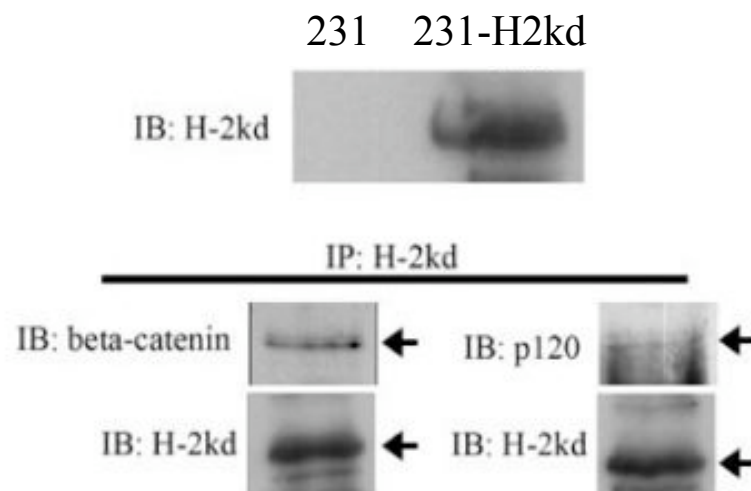

Supplement: Additional file 2 — β- and p120-catenin are sequestered by the Ecad/H2kd fragment. A) β- or p120-catenin, left panel, green; H2kd, middle panel, red; merge, right panel, yellow. In the merged images, the catenins colocalize with the H2kd molecules. B) β-catenin staining of 231, 231-Ecad and MCF7 cells. β-catenin is localized at the membrane in 231-Ecad and MCF7 cells but in the cytoplasm in 231 cells. C) Transfected MDA-231 cells express the H2kd fragment. When 231-H2kd whole cell lysates are probed with an H2kd antibody and immunoprecipitated, both beta- and p120 catenins coimmunoprecipitate as determined by western blot. [file 1476-4598-9-179-S2.PDF]

A

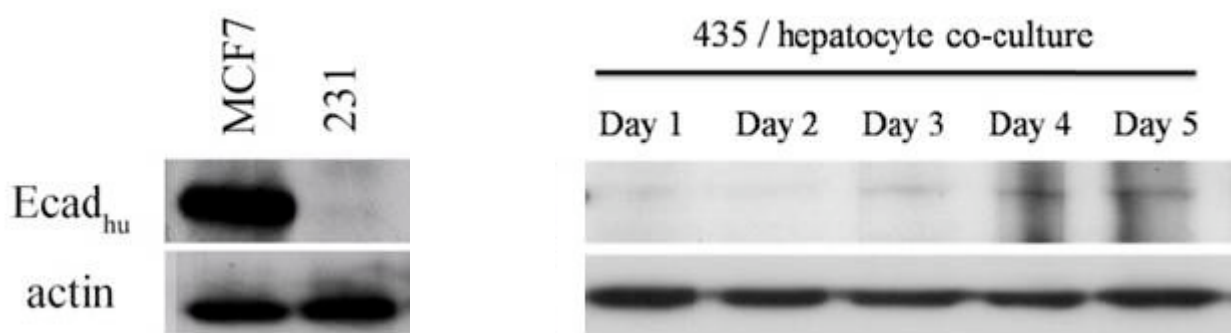

B

Total cancer cell and hepatocyte DNA isolated from coculture

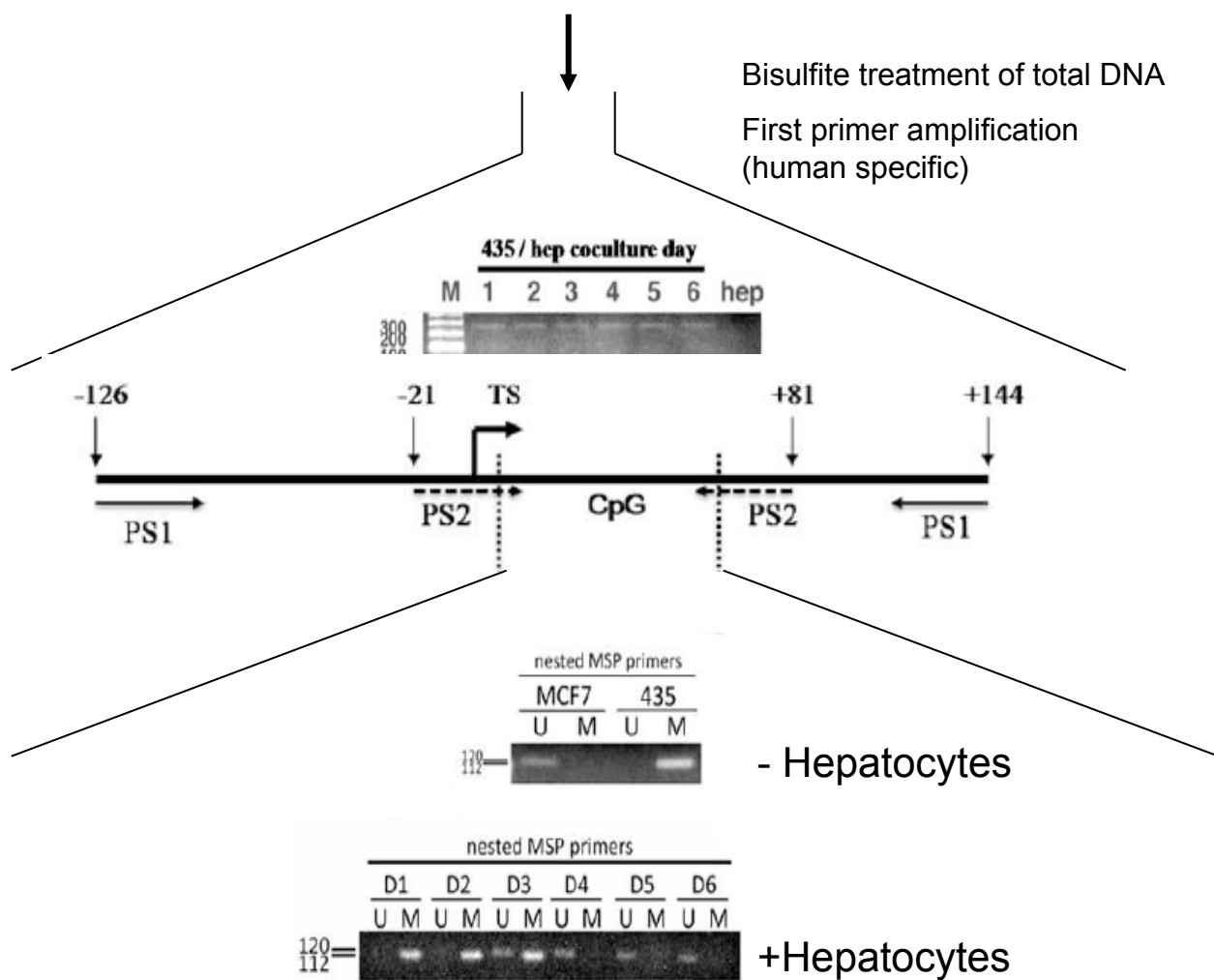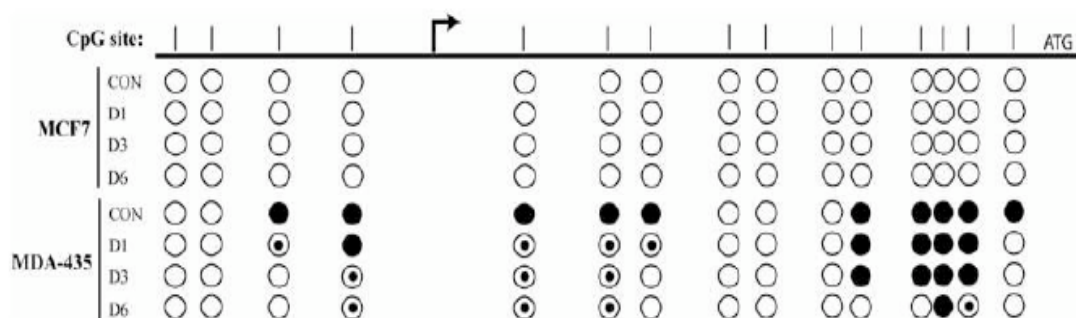

Supplement: Additional file 3 — MDA-MB-435 cells re-express E-cadherin. A) Immunoblot of MDA-MB-435 cells cultured with hepatocytes for 6 days and probed with an E-cadherin antibody. B) Methylation-specific PCR of MDA-MB-435/hepatocyte samples reveals loss of methylation of the E-cadherin promoter. [file 1476-4598-9-179-S3.PDF]
